# Supplementary material for: Clinical Impact of Prospective Whole Genome Sequencing in Sarcoma Patients
Source: Cancers (Basel). 2022 Jan 16;14(2):436. doi: 10.3390/cancers14020436 (PMC8773512; doi:10.3390/cancers14020436)
Supplement: Supplementary file 1 [file Supplementary Figure S1 - Detailed description pathology revision cases.pdf › Supplementary Figure S1 - Detailed description pathology revision cases.pdf]

Supplementary Figure S1. Morphology and immunohistochemistry of all revision cases.

Patient 8
Diagnosis prior to WGS: carcinoma of unknown primary with HER2 overexpression
Diagnosis after WGS: synovial sarcoma

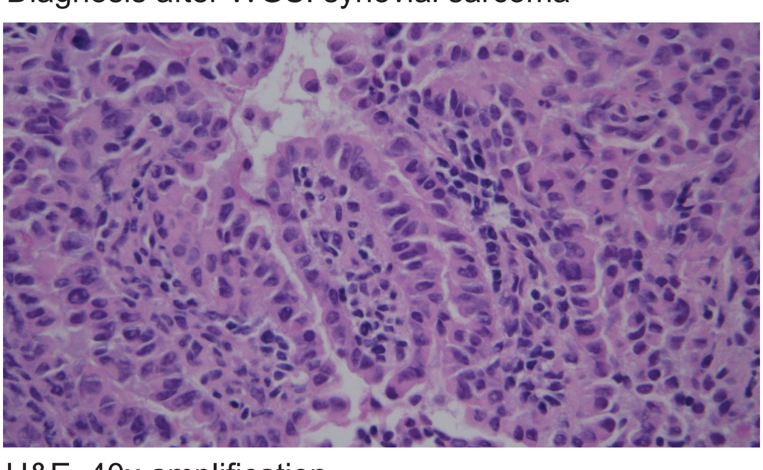
H&E, 40x amplification

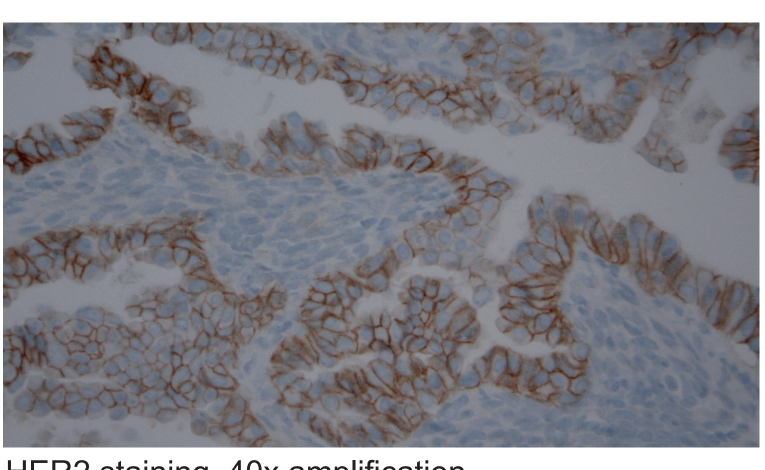
HER2 staining, 40x amplification

Immunohistochemistry
Positive: CAM5.2, CD99, CK7, synaptofysin, TLE1
Partially positive: BCL2, CDX2, HER2
Negative: S100, AR, chromogranin, CK20, napsine A, p16, p53, p63, thyreoglobulin, TTF-1

Patient 44
Diagnosis prior to WGS: alveolar rhabdomyosarcoma
Diagnosis after WGS: spindle cell/sclerosing rhabdomyosarcoma

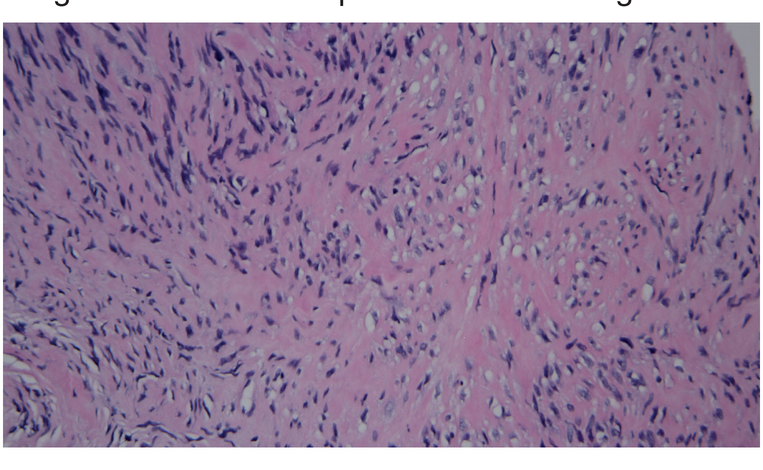
H&E, 20x amplification

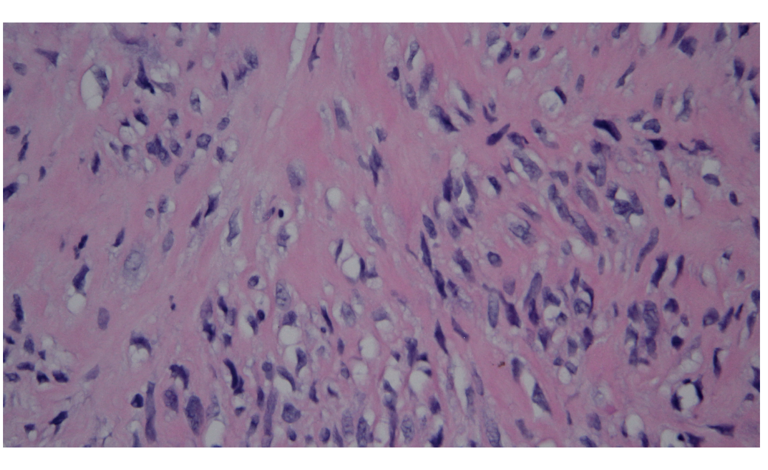
H&E, 40x amplification

Immunohistochemistry
Positive:aSMA, H3K27me3
Partially positive: CD10, Myf4, desmine, myogenin
Negative: p53, p63, S100, melanA, ALK, caldesmon-H, CD34, MUC4

Patient 45
Diagnosis prior to WGS: embryonal rhabdomyosarcoma
Diagnosis after WGS: spindle cell/sclerosing rhabdomyosarcoma

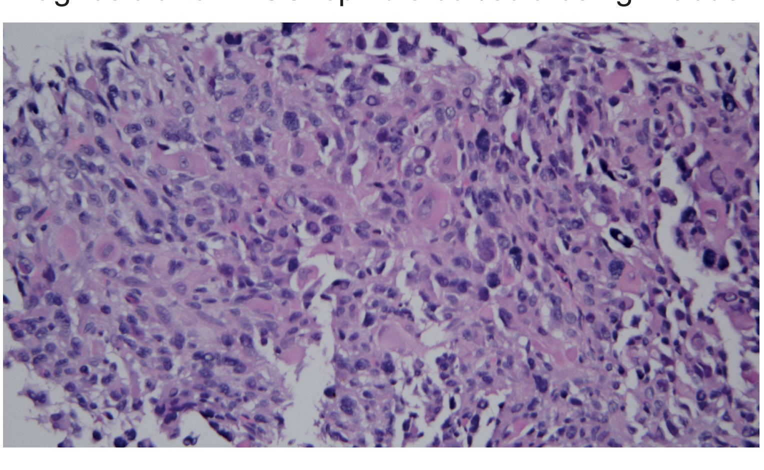
H&E, 20x amplification

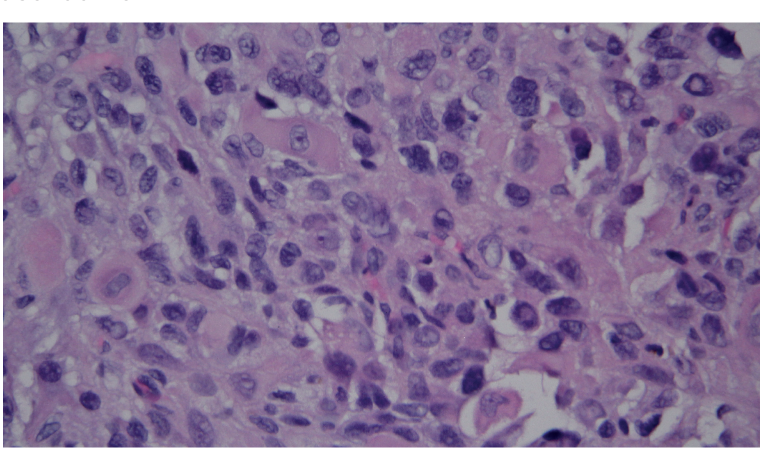
H&E, 40x amplification

Immunohistochemistry
Positive: ALK, desmine, H3K27me3, MYOD1
Partially positive:
Negative: CD3, CD20, CD30, CD45, CD138

Patient 46
Diagnosis prior to WGS: undifferentiated pleiomorphic sarcoma
Diagnosis after WGS: spindle cell/sclerosing rhabdomyosarcoma

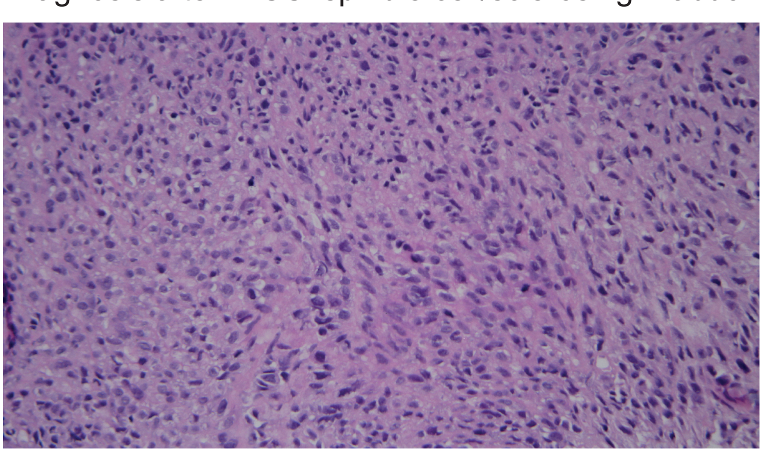
H&E, 20x amplification

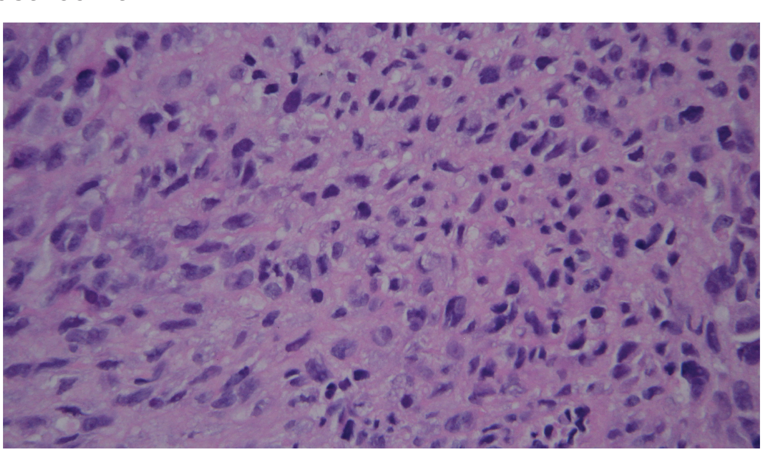
H&E, 40x amplification

Immunohistochemistry
Positive: ALK, LCA, CD1a, CD56, CD163, SATB2
Partially positive: S100, CD31, aSMA, calponine, desmine, CK AE1/3, CD3, CD20
Negative: myogenin, Myf4, CAM5.2, EMA, CD34, MART-1, MDM2, HMB45, melanA, E-cadherin, CD99

Patient 47
Diagnosis prior to WGS: sarcomatoid mesothelioma vs. sarcoma
Diagnosis after WGS: sarcoma

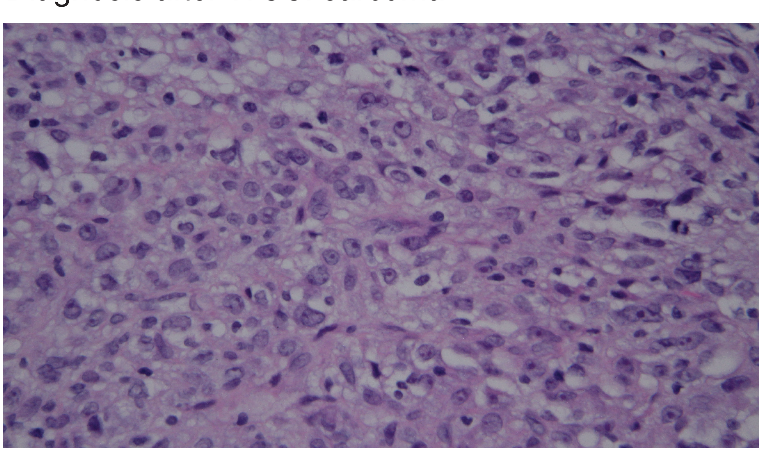
H&E, 40x amplification

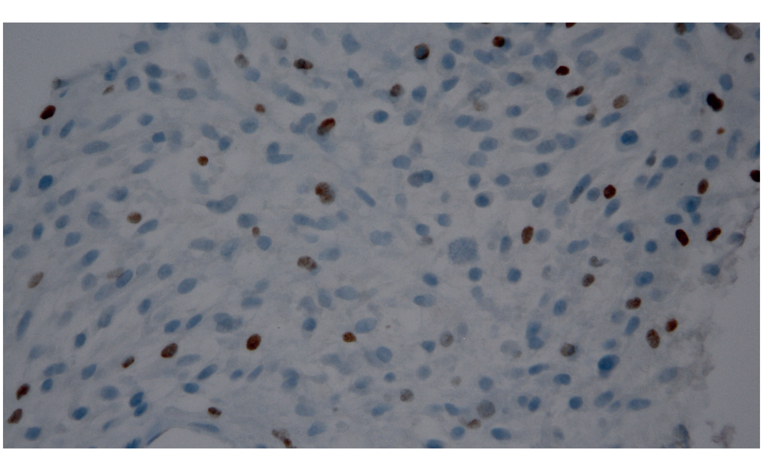
Negative H3K27me3 staining, 40x amplification.
NB. Lymphocytes show normal H3K27me3 expression

Immunohistochemistry
Positive: BAP1, calretinin, CD30, CD31, CD99, D2-40, vimentin
Partially positive: aSMA, CD68
Negative: ALK, CAM5.2, CD21, CD23, CEA, CK AE1/3, CK7, desmine, EMA, Myf4, p53, p63, S100, SOX10, STAT6, TLE1, WT-1, INI, H3K27me3

Patient 54
Diagnosis prior to WGS: wild type GIST
Diagnosis after WGS: KIT mutated GIST

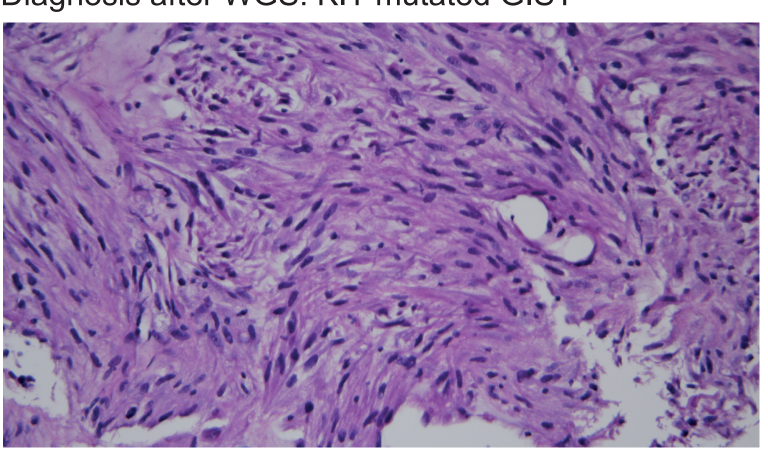
H&E, 20x amplification

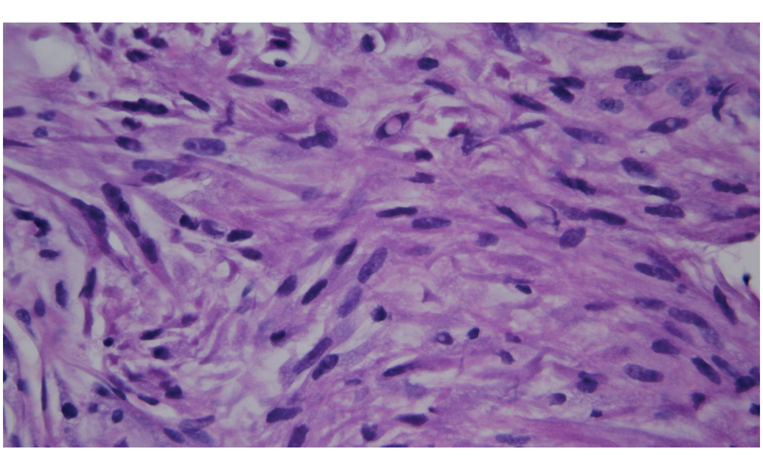
H&E, 40x amplification

Immunohistochemistry
Positive: DOG1, SDH
Partially positive:
Negative:

Patient 57
Diagnosis prior to WGS: Ewing sarcoma
Diagnosis after WGS: Myoepithelial carcinoma of soft tissue

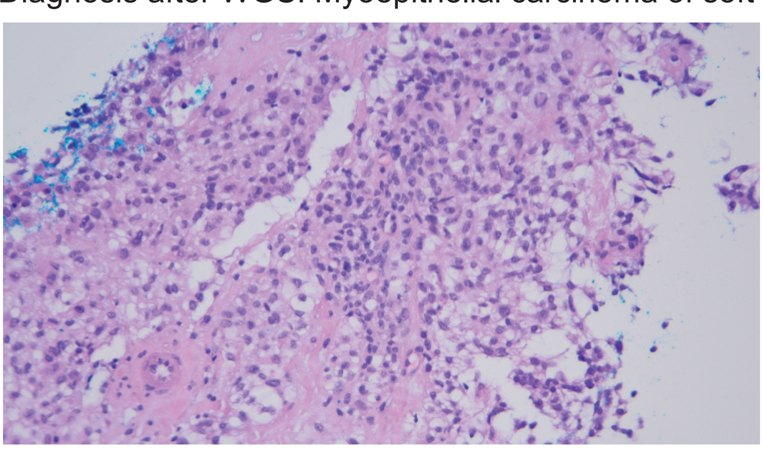
H&E, 20x amplification

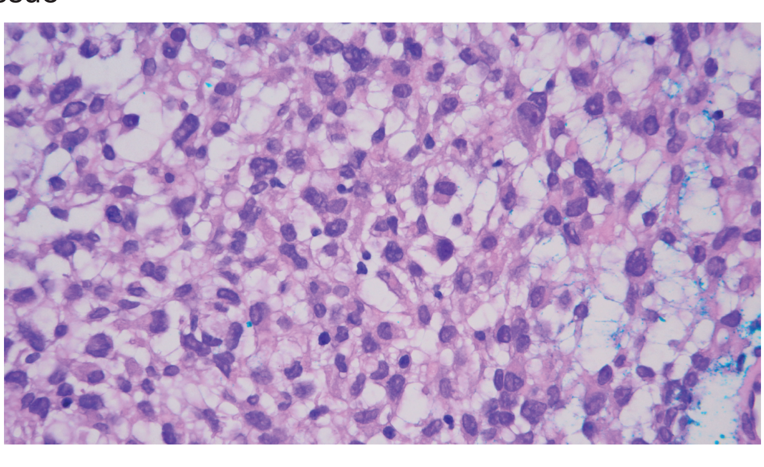
H&E, 40x amplification

Immunohistochemistry
Positive: S100, CK AE1/3, PAX8, vimentin, CD99
Partially positive: CAM5.2, EMA
Negative: LCA, TTF-1, CD163, thyreoglobulin, ER, CD1a, MelanA, HMB45, RCC, SOX10, SMA, calponine, p63, ERG, BRAF VE1

Patient 63
Diagnosis prior to WGS: dedifferentiated liposarcoma
Diagnosis after WGS: radiotherapy-associated secondary primary

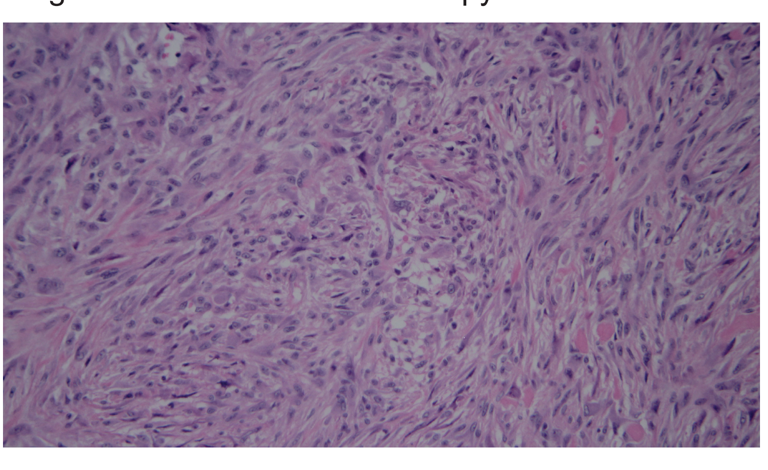
H&E, 20x amplification of initial tumor (DD-LPS)

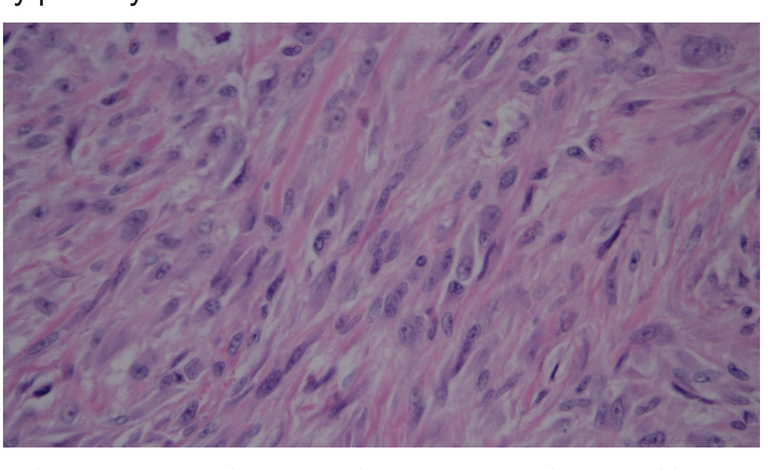
H&E, 40x amplification of initial tumor (DD-LPS)

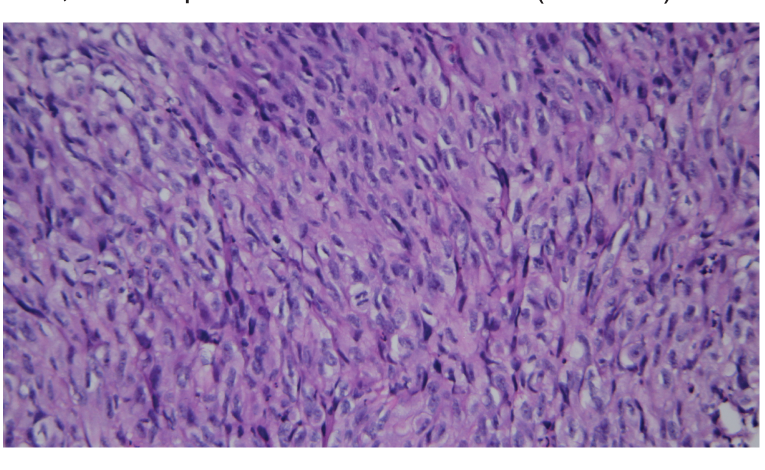
H&E, 20x amplification of second primary tumor

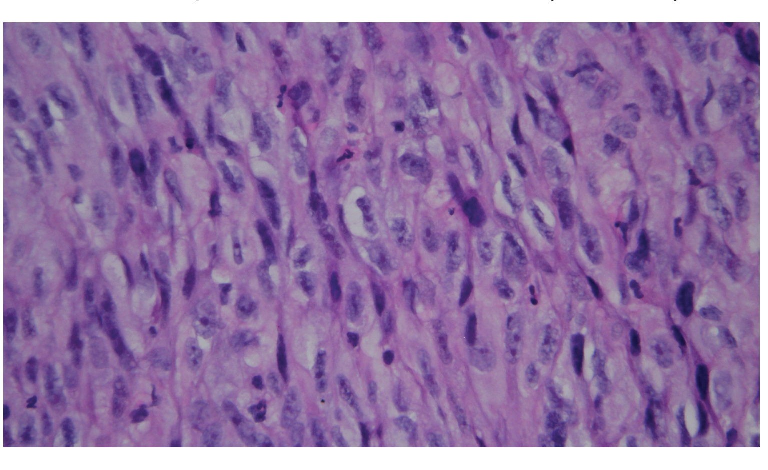
H&E, 40x amplification of second primary tumor

Immunohistochemistry (second tumor)
Positive: RB1
Partially positive: MDM2, CDK4
Negative: p16, p53

Patient 80
Diagnosis prior to WGS: carcinoma of unknown primary
Diagnosis after WGS: Desmoplastic Small-Round-Cell Tumor

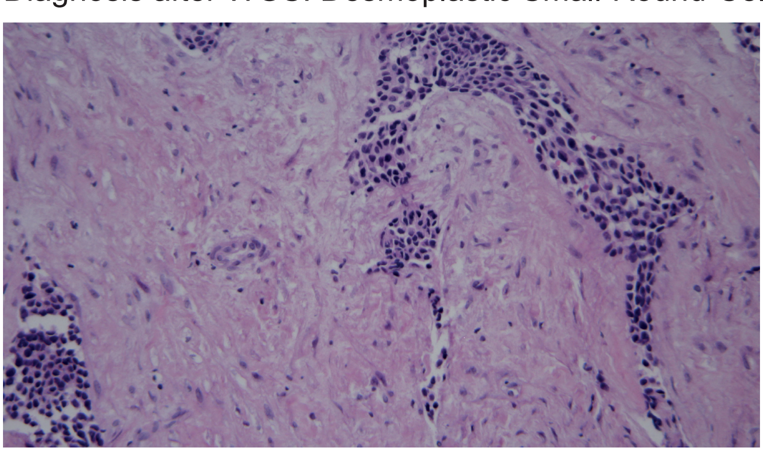
H&E, 20x amplification

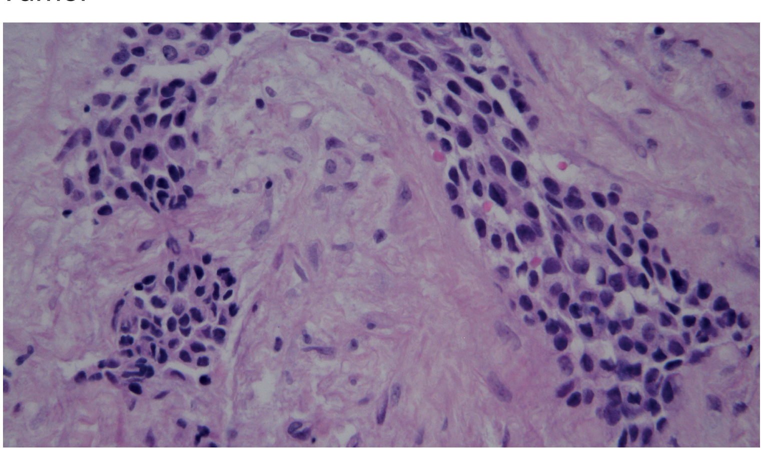
H&E, 40x amplification

Immunohistochemistry
Positive: CK AE1/3, CD56, vimentin, RB1, p53, EMA, Epcam, CD99, BAP1, INI1, CAM5.2
Partially positive: NKX3.1, SATB2, calretinin
Negative: CA125, CD10, CD117, CD20, CD3, CD34, CDX2, CEA, chromogranin, CK20, CK7, desmin, DOG1, ERG, GATA-3, glypica-3, hepar, LCA, melanA, napsine, NUT, OCT3/4, p16, p40, p63, PAX-8, PLAP, PMSA, PSA, PSAP, synaptophysin, TTF-1, WT1, XO10

Patient 81
Diagnosis prior to WGS: Malignant peripheral nervous sheath tumor
Diagnosis after WGS: Melanoma

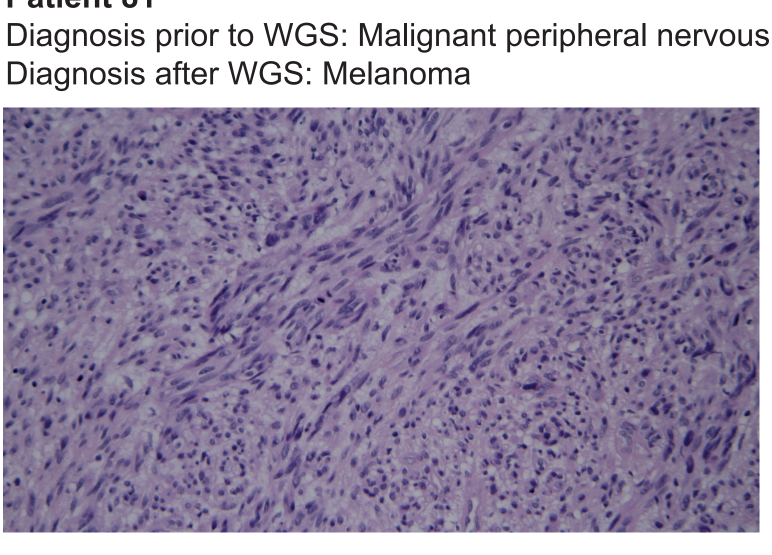
H&E, 20x amplification

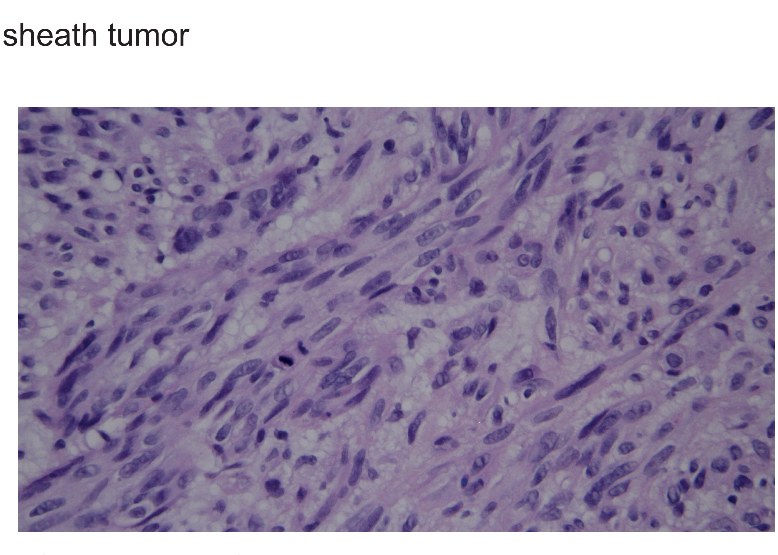
H&E, 40x amplification

Immunohistochemistry
Positive: SOX10, TLE1, INI1, p16, S100, H3K27me3
Partially positive: CK7, EMA
Negative: CK AE1/3, desmine, HMB45, MelanA, STAT6, CD34, BRAF VE1, p53

Patient 82
Diagnosis prior to WGS: Melanoma vs. sarcoma
Diagnosis after WGS: Melanoma

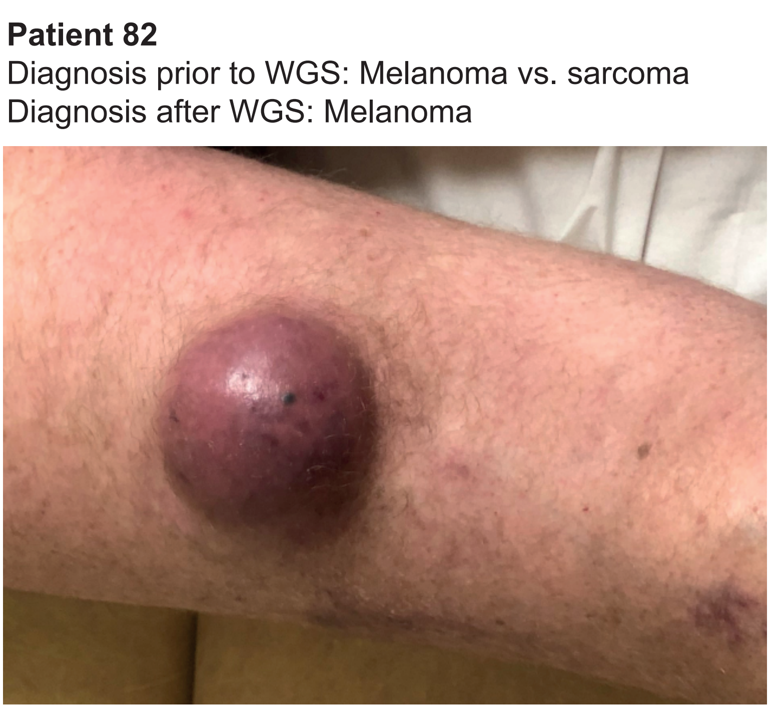
Patient 82 presented with a large lesion on the thigh.Due to the physical appearance and lack of distinctive immunohistochemical staining, it was not possible to distinghuish between sarcoma and melanoma

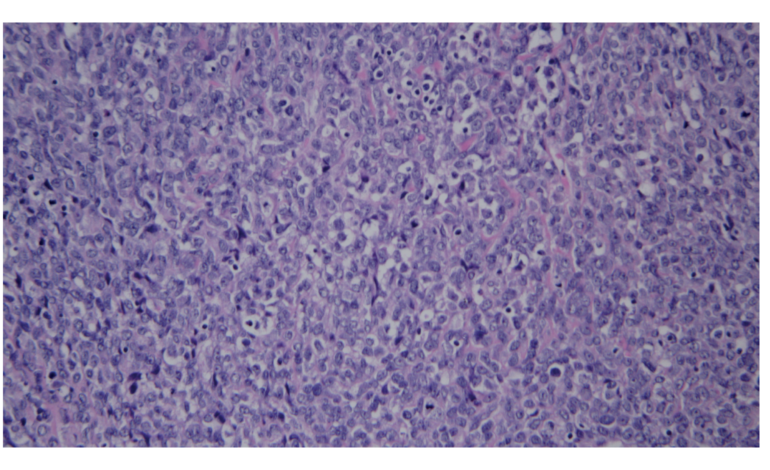
H&E, 20x amplification

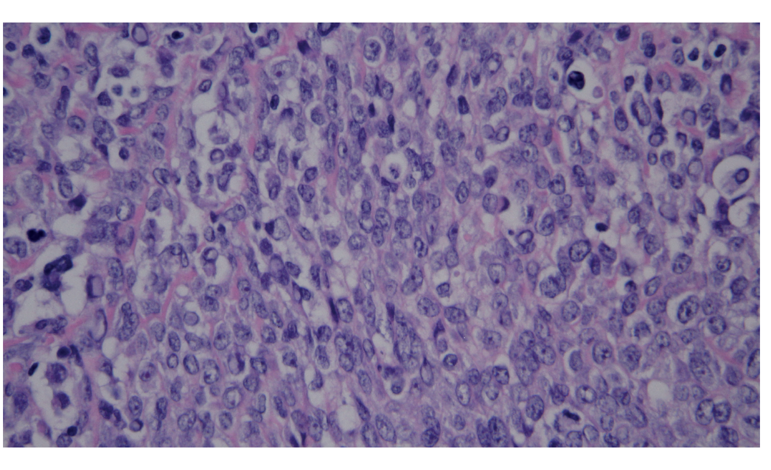
H&E, 40x amplification

Immunohistochemistry
Positive: vimentine, INI1, CD99, PAS, BRAF
Partially positive: SMA, CD34, EMA, CK AE1/3, STAT6
Negative: MDM2, H3K27me3, OCT3/4, LCA, DOG1, CD21, synaptophysin, PAX5, MITF, CD5, CD138, CD23, CD2, CD68, Myf4, MelanA, p53, CD3, CD300, CD20, CDK4, HMB45, ERG, desmine, S100, SOX10, TLE1

Patient 83
Diagnosis prior to WGS: Interdigitating dendritic cell sarcoma
Diagnosis after WGS: Melanoma

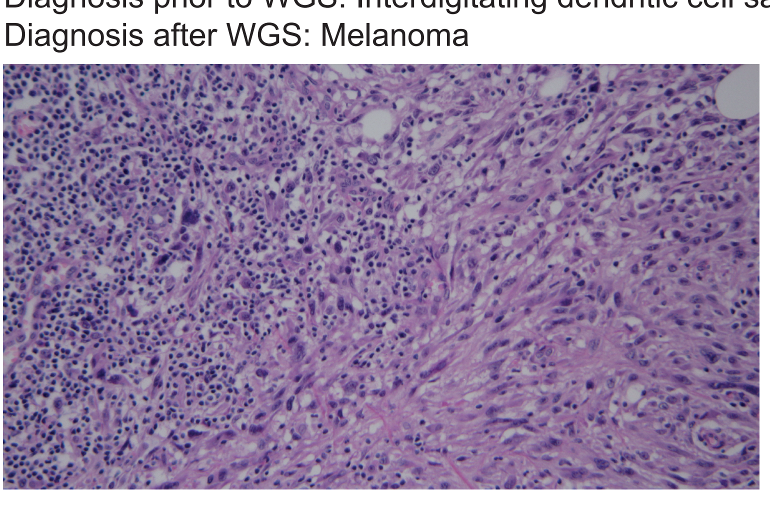
H&E, 20x amplification

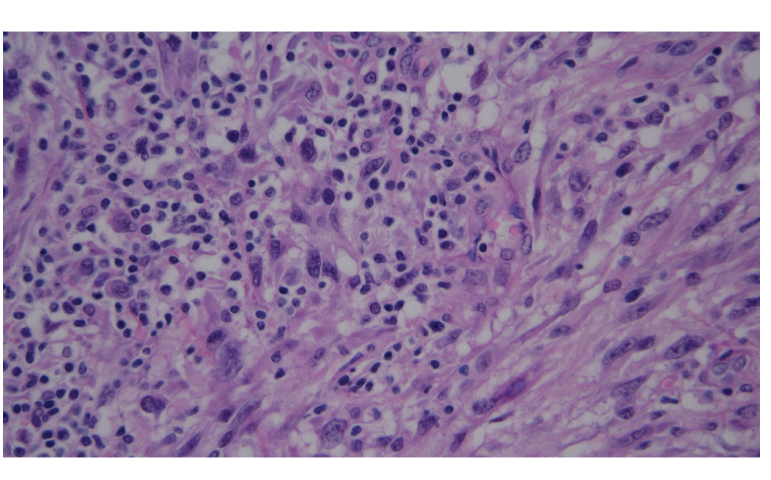
H&E, 40x amplification

NB. This patient had a history of concurrent chronic lymphocytic leukemia

Immunohistochemistry
Positive: p53, S100, SOX10, aSMA, CD68, INI1
Partially positive: CD23, PAX5, CD20, CD5
Negative: CD21, CD1a, CD34, CK AE1/3, HMB45, MelanA, MITF, BRAF VE1, desmine, caldesmon-H, EMA
