# Supplementary material for: Clinical Impact of Prospective Whole Genome Sequencing in Sarcoma Patients
Source: Cancers (Basel). 2022 Jan 16;14(2):436. doi: 10.3390/cancers14020436 (PMC8773512; doi:10.3390/cancers14020436)
Supplement: Supplementary file 1 [file cancers-14-00436-s001.zip › Supplementary Figure S2 - Schematic overview of a large KIT exon 11 deletion in a patient with a gastrointestinal stromal tumor..pdf]

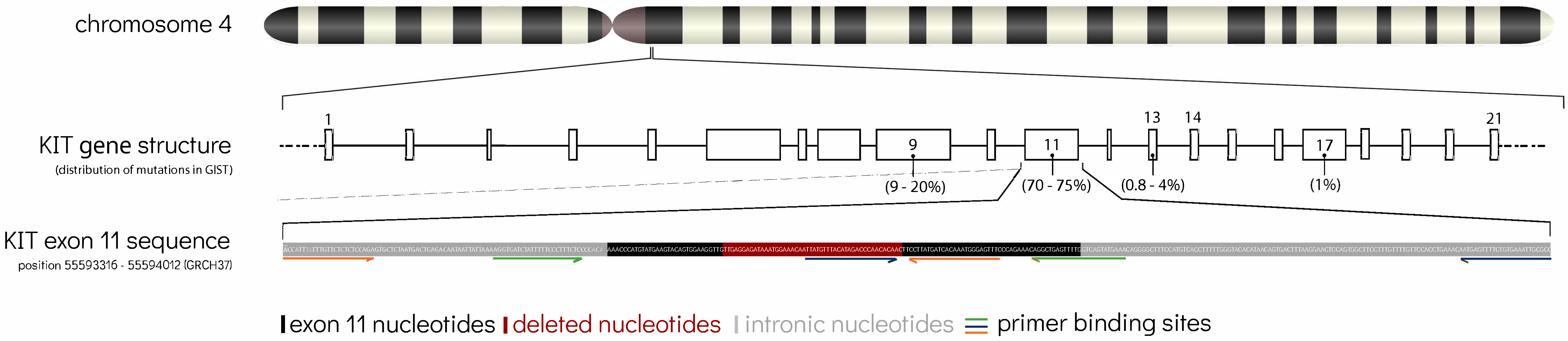

Supplementary Figure S2. Schematic overview of a large KIT exon 11 deletion in a patient with a gastrointestinal stromal tumor. The deletion of 51 base pairs remained undetected with repetitive capture-based Ampliseq panel sequencing despite the use of three different primers. For two primers, an extensive proportion of the read length was somatically deleted, and the complete read was filtered out by the analytical pipeline to prevent false-positive deletion calling. For one of the primers, the primer binding sites overlapped with the deletion (blue primer).
