# Supplementary material for: Clinical Impact of Prospective Whole Genome Sequencing in Sarcoma Patients
Source: Cancers (Basel). 2022 Jan 16;14(2):436. doi: 10.3390/cancers14020436 (PMC8773512; doi:10.3390/cancers14020436)
Supplement: Supplementary file 1 [file cancers-14-00436-s001.zip › Supplementary Figure S3 - Timeline plots of patients with diagnostic revisions with direct therapy implications.pdf]

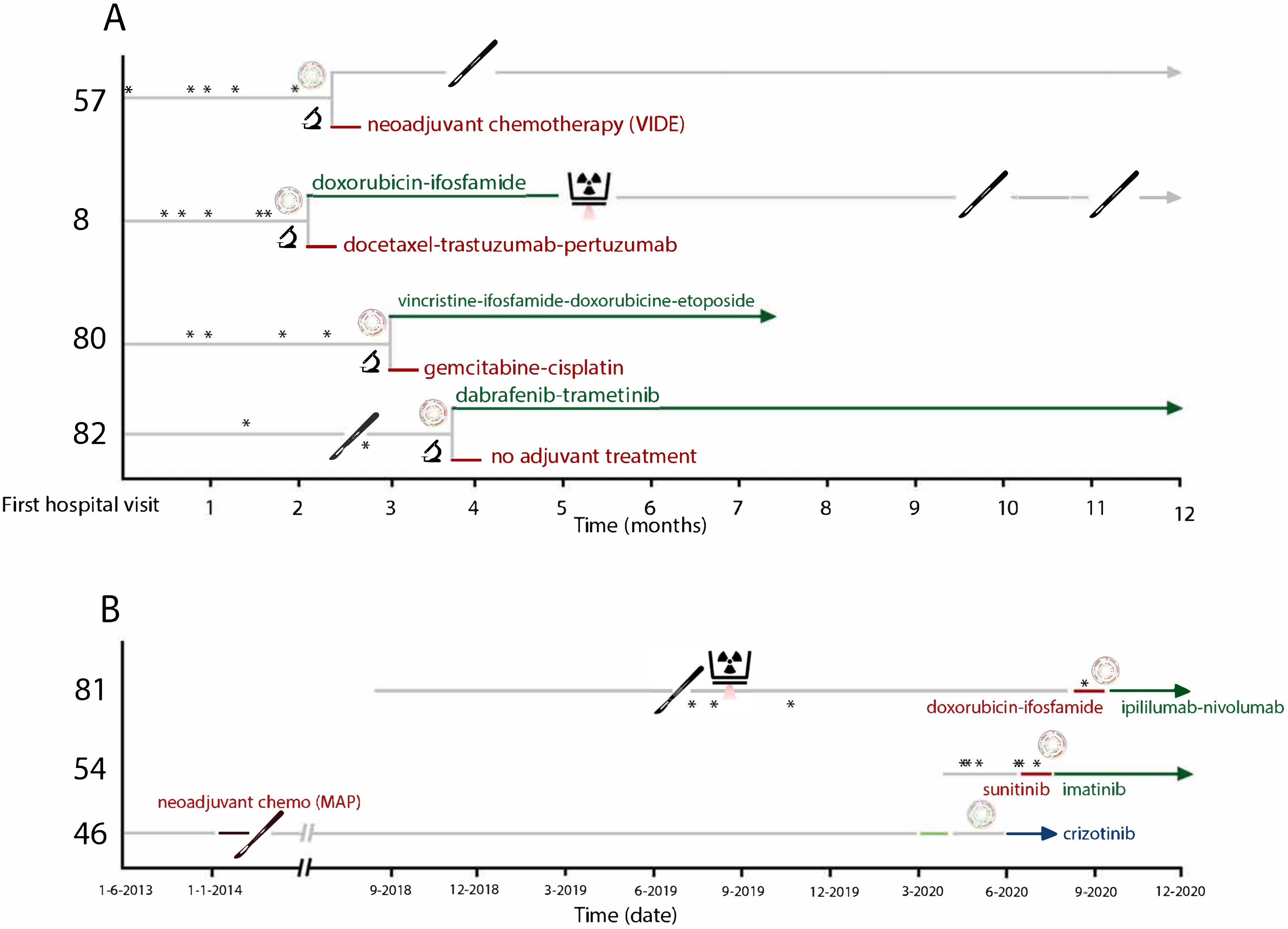

Supplementary Figure S3. Timeline plots of patients with diagnostic revisions with direct therapy implications. For five patients (A), treatment could be optimized after identification of the true tumor type. Overtreatment with inappropriate treatment choices was prevented in three patients, while treatment could be optimized for one with the addition of adjuvant dabrafenib-trametinib. Treatment depicted in dark green represents the treatment that started after the WGS result, including clinical follow-up, the treatment in red represents the treatment of choice under misdiagnosis. One patient did not start systemic treatment. Three patients (B) already received treatments in the past, before the WGS result (red treatment lines). Treatment choices were adjusted after WGS result for two patients, one patient (663) received targeted therapy for an ALK fusion detected with WGS analysis (blue line). The light green line represents treatment with pazopanib, a viable therapy option for the revised diagnosis.
* = separate pathological assessment (either new tumor material, or a revision of existing material by an independent pathologist).
